# An Artemisinin-Derived Dimer Has Highly Potent Anti-Cytomegalovirus (CMV) and Anti-Cancer Activities

**DOI:** 10.1371/journal.pone.0024334

**Published:** 2011-08-31

**Authors:** Ran He, Bryan T. Mott, Andrew S. Rosenthal, Douglas T. Genna, Gary H. Posner, Ravit Arav-Boger

**Affiliations:** 1 Department of Pediatrics, Johns Hopkins University School of Medicine, Baltimore, Maryland, United States of America; 2 Department of Chemistry, School of Arts and Sciences, The Johns Hopkins University, Baltimore, Maryland, United States of America; 3 Malaria Research Institute, Bloomberg School of Public Health, Johns Hopkins University School of Medicine, Baltimore, Maryland, United States of America; The Scripps Research Institute, United States of America

## Abstract

We recently reported that two artemisinin-derived dimers (dimer primary alcohol 606 and dimer sulfone 4-carbamate 832-4) are significantly more potent in inhibiting human cytomegalovirus (CMV) replication than artemisinin-derived monomers. In our continued evaluation of the activities of artemisinins in CMV inhibition, twelve artemisinin-derived dimers and five artemisinin-derived monomers were used. Dimers as a group were found to be potent inhibitors of CMV replication. Comparison of CMV inhibition and the slope parameter of dimers and monomers suggest that dimers are distinct in their anti-CMV activities. A deoxy dimer (574), lacking the endoperoxide bridge, did not have any effect on CMV replication, suggesting a role for the endoperoxide bridge in CMV inhibition. Differences in anti-CMV activity were observed among three structural analogs of dimer sulfone 4-carbamate 832-4 indicating that the exact placement and oxidation state of the sulfur atom may contribute to its anti-CMV activity. Of all tested dimers, artemisinin-derived diphenyl phosphate dimer 838 proved to be the most potent inhibitor of CMV replication, with a selectivity index of approximately 1500, compared to our previously reported dimer sulfone 4-carbamate 832-4 with a selectivity index of about 900. Diphenyl phosphate dimer 838 was highly active against a Ganciclovir-resistant CMV strain and was also the most active dimer in inhibition of cancer cell growth. Thus, diphenyl phosphate dimer 838 may represent a lead for development of a highly potent and safe anti-CMV compound.

## Introduction

Infection with CMV, a member of the herpesvirus family, is common in humans. Seroprevalence rates increase with age, reaching 90% in individuals older than 80 years [1]. The virus establishes lifelong persistent infection, which usually remains asymptomatic. In immunocompromised hosts such as transplant recipients and patients with AIDS, CMV infection is associated with significant morbidity and mortality [2], [3]. CMV is also the most common congenitally-acquired infection causing mental retardation and deafness in congenitally-infected children [4]. Recently, the detection of CMV in immunocompetent individuals has been linked with outcomes of several syndromes including sepsis, pulmonary complications in patients in intensive care-units, and in a brain tumor, glioblastoma multiforme [5]–[7]. Although the direct role of CMV in these syndromes is unclear, virus replication may contribute to their natural history, and the role of anti-CMV therapy in these conditions is currently being investigated.

The available systemic anti-CMV drugs act by targeting the viral DNA polymerase. These compounds effectively suppress CMV replication, but their use is associated with considerable toxicities to the bone marrow (Ganciclovir-GCV) and kidneys (Foscarnet and Cidofovir) [8], [9] and the emergence of drug-resistant mutants during prolonged courses of therapy [9], [10]. Thus, new compounds with low toxicity and ideally with a distinct mechanism of CMV inhibition are needed for CMV therapy.

The artemisinin-derived monomer artesunate was originally reported to inhibit CMV replication *in vitro* and *in vivo* [11], [12]. Recently, we reported on two artemisinin-derived dimers with significantly more potent activity against CMV replication *in vitro* as compared to artemisinin-derived monomers [13]. Artemisinin monomers are currently the drugs of choice for malaria therapy [14]. In addition, both artemisinin monomers and dimers were shown to possess anti-cancer activities [15]–[17]. The potent anti-CMV activity of two artemisinin-derived dimers [13] prompted us to evaluate a series of newly-synthesized artemisinin-derived dimers. We report on the anti-CMV and anti-cancer activities of the most potent compounds in this investigation.

## Results

### A comparison of anti-CMV activity of 17 artemisinin derivatives

We previously reported on the anti-CMV activity of four artemisinin monomers (artemisinin, artesunate, artemether, and artefanlide) and two artemisinin-derived dimers (dimer primary alcohol 606 and dimer sulfone 4-carbamate 832-4) [13]. We now tested one new artemisinin-derived monomer and 10 additional new artemisinin-derived dimers and compared their anti-CMV activities to those of previously tested compounds. The abbreviated names of the 17 compounds and their molecular weights are listed in Table 1. In this report, each compound is referred to by its molecular weight. For example, compound 606 refers to the dimer primary alcohol, and 832-4 refers to dimer sulfone 4-carbamate (Table 1). Sulfone carbamate 551 is the monomeric version of dimer sulfone carbamate 832-4. The chemical structure of dimer sulfone 4-carbamate 832-4 prevents it from being catabolized into monomer sulfone 4-carbamate 551. Compound 574, which is the deoxy version of 606, was chosen for testing because the anti-malarial and anti-cancer activities of artemisinins are at least partially endoperoxide bridge-dependent [18], [19].

**Table 1:** List of artemisinin-derived monomers (first 5 compounds) and dimers evaluated for anti-CMV activity, and their molecular weight (MW).

Compound	MW
Artemisinin	282
Artemether	298
Artesunate	384
Artefanilide (ref 33)	433
Art-PrSO_2_Ph-4-CH_2_OC(O)NMe_2_	551
Deoxy-Dimer-isobu-OH	574
Dimer isobu-OH	606
Dimer isobu-COOH	620
5C Dimer OH	644
Dimer isobu-SO_2_Ph-4-CH_2_OH	760
Dimer isobu-SPh-3-CH_2_OC(O)NMe_2_	800-3
Dimer isobu-SPh-4-CH_2_OC(O)NMe_2_	800-4
5C Dimer OC(O)Ph-4-SO_2_Me	826
Dimer isobu-SO_2_Ph-4-CH_2_OC(O)NMe_2_	832-4
Dimer isobu-SO_2_Ph-3-CH_2_OC(O)NMe_2_	832-3
Dimer isobu-OP(O)(OPh)_2_	838
5C Dimer OC(O)CH_2_N(Boc)Bn	891

**Figure 1:**
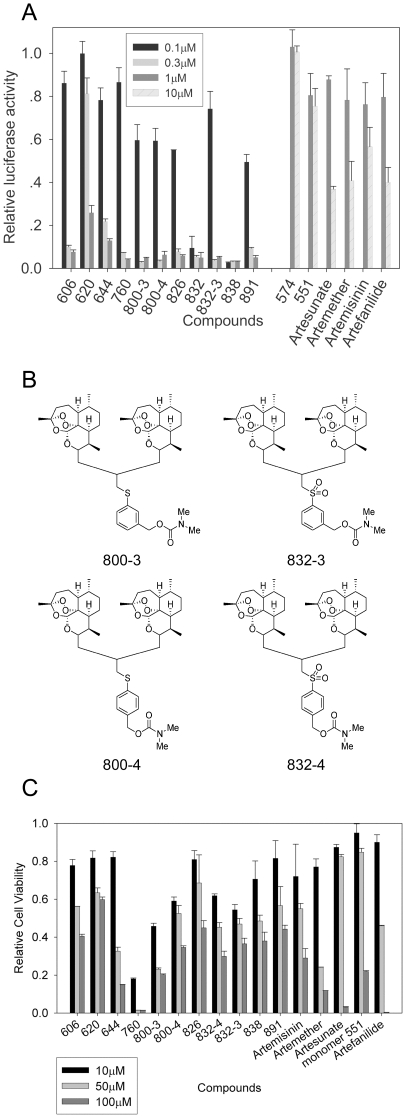
Anti-CMV activity and cytotoxicity of artemsinin-derived compounds. **A**: Relative luciferase activity of artemsinin-derived dimers (left), deoxy dimer 574, and artemisinin monomers (right). Infected and treated HFFs were collected 72 hpi and washed once with PBS. The lysates were assayed for luciferase activity using the Luciferase Assay Kit (Promega, Madison, WI). **B**. Chemical structure of analogs of dimer sulfone 4-carbamate 832-4. Depicted are two sulfide carbamate analogs (800-3 and 800-4) and one sulfone 3-carbamate analog (832-3) of sulfone 4-carbamate 832-4. **C**: Cytotoxicity of ten artemisinin-dimers and five artemisinin-monomers in HFF. HFFs treated with dimers/monomers at the indicated concentrations were collected at 72 and washed once with PBS. The lysates were assayed for cell viability using CellTiter-Glo Luminescent Cell Viability Assay Kit on GloMax®-Multi+ Detection System (Promega) according to manufacturer's instructions.

The two previously tested artemisinin-derived dimers had potent anti-CMV activity at concentrations of 1 µM or lower, while the artemisinin-derived monomers achieved a similar degree of CMV inhibition only at concentrations higher than 10 µM [13]. Based on these data, all new compounds were initially screened for anti-CMV activities. The most active compounds were then tested in detail for their anti-CMV activities. All dimers were initially evaluated at concentrations of 1 µM, 0.3 µM, and 0.1 µM. The deoxy dimer 574 and monomer sulfone carbamate 551 were screened at 1 µM and 10 µM. At 1 µM, the dimers displayed potent inhibition of late pp28 gene expression (which highly correlates with plaque reduction) [20] measured by luciferase activity, but the deoxy dimer 574 and monomer sulfone carbamate 551 did not (Fig. 1A). Several dimers were also effective at 0.3 µM, but only two dimers, sulfone 4-carbamate 832-4 and diphenyl phosphate 838, were highly inhibitory at 0.1 µM. Three structural analogs of sulfone 832-4 were synthesized (832-3, 800-3 and 800-4) varying the oxidation state and the position of the sulfur atom on the aromatic ring (Fig. 1B). Though the meta-sulfide (800-3), meta-sulfone (832-3), and para-sulfide (800-4) were active against CMV replication at 0.3 µM, unlike para-sulfone 832-4 they were not active at 0.1 µM (Fig. 1A). Therefore, the exact placement and oxidation state of the sulfur atom contributes to its anti-CMV activity.

We next screened for cellular toxicity of the dimers and monomers using CellTiter-Glo Luminescent Cell Viability assay at concentrations of 10 µM, 50 µM and 100 µM. For most dimers (except 644 and 760), 50% cellular cytotoxicity (CC_50_) ranged from 50 to 100 µM (Fig. 1C). Monomers overall displayed similar cytotoxicity pattern, but were significantly more toxic than dimers at 100 µM (P = 0.03 by two-tailed t-test). Dimers 832-4 and 838 had the best selectivity index (SI), which is the ratio of CC_50_ to EC_50_, and were therefore selected for further detailed analysis of anti-CMV activity.

### Structure-activity relationship and cell toxicity of dimers 832-4 and 838 and their parent dimers 760 and 606

**Figure 2:**
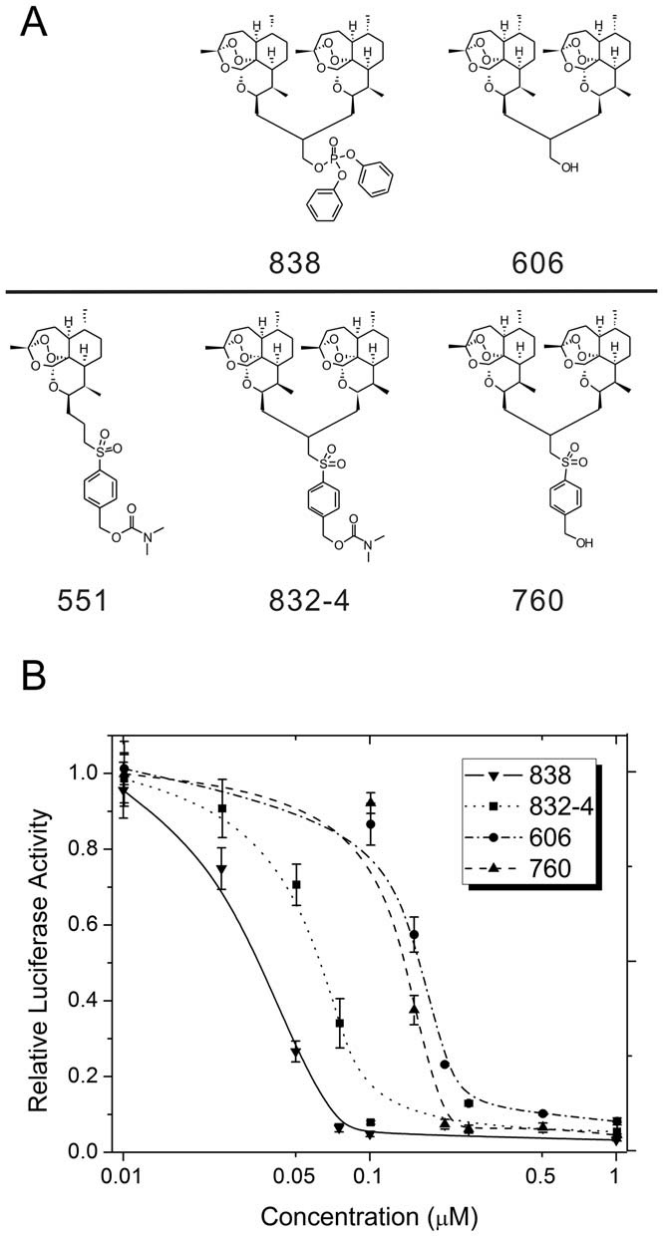
Chemical structure and anti-CMV activity of the two most potent artemisinin-derived dimers and their corresponding parent compounds. **A**: Chemical structures of the two most potent artemisinin-derived dimers 838 and 832-4 and their corresponding parent alcohols 606 and 760. Monomer 551 is depicted on the left of dimer 832-4. The sulfone benzylic dimethyl carbamate unit is the same in monomer 551 as in dimer 832-4. Therefore, the enhanced anti-CMV activity of the dimer 832-4 over monomer 551 is not due to any difference in the sulfone carbamate portion of the molecule. **B**: Detailed anti-CMV activity of dimers 832-4 and 838, and their respective parent compounds 760 and 606. CMV-infected HFF were treated with dimers 832-4 and 838 at the indicated concentrations. 72 hpi luciferase activity was determined in the lysates of infected cells. Luciferase values represent the mean and SD of data derived from at least four independent experiments performed in triplicates. The curve fitting toolbox, Matlab software (v7.5), Mathworks (Natick, MA) was used to determine EC_50_ values using a four-parameter logistic regression.

A detailed comparison of anti-CMV activity and toxicity in non-cancer primary HFF cells was next performed with the most potent dimers, 832-4 and 838 (Fig. 2A). A dose-dependent increase in anti-CMV activity was observed from 0.01 µM to 1 µM for both dimers 832-4 and 838 (Fig. 2B, Table 2). Cellular toxicities, measured at 1 µM to 100 µM (Table 2), were not observed at concentrations of complete CMV inhibition. The EC_50_, CC_50_ and SI are shown in Fig. 2B and Table 2. Dimers 832-4 and 838 had a high SI, but 838 was the best compound. The parent alcohols from which 832-4 and 838 were synthesized, 760 and 606, respectively, were less effective than their derivatives in CMV inhibition. For the 606-838 pair the difference in SI was 5-fold, and for the 760-832-4 pair, 30-fold (Fig. 2A, 2B). Although the dimer sulfone 4-carbamate 832-4 and the monomer sulfone 4-carbamate 551 have identical sulfone 4-carbamate functional groups (Fig. 2A), dimer 832-4 was at least 150 fold more effective in CMV inhibition than monomer 551.

**Table 2:** EC_50_, CC_50_, selectivity index (SI) and *m* (slope) for the most potent dimers, artesunate and GCV.

Compounds	EC_50_ (µM)	CC_50_ (µM)	SI	Slope
832-4	0.07±0.004	57.5±2.9	909.8±81	3.6±0.7
838	0.04±0.003	55.8±2.8	1472±127	3.9±0.2
760	0.14±0.008	4.9±0.2	34.5±2	4.0±1.0
606	0.16±0.008	48.1±2.6	292±21	3.5±0.7
artesunate	6.6±0.4	71.7±4	11±0.9	1.1±0.02
GCV	2.7±0.1	247±33.4	96±14	1.3±0.06

Dimer alcohol 760 is the parent compound of 832-4 dimer sulfone carbamate. Dimer alcohol 606 is the parent compound of 838 dimer diphenyl phosphate.

### Inhibition of a GCV-resistant CMV strain by dimers 832-4 and 838

**Figure 3:**
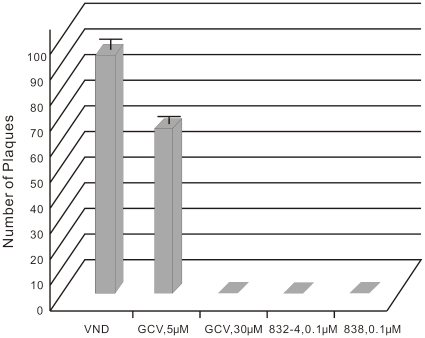
Activity of 832-4, 838 and GCV against a GCV-resistant CMV strain. A plaque reduction assay was used to quantify the inhibition of the GCV-resistant CMV. CMV-infected HFF were treated with 832-4, 838 and GCV at the indicated concentrations. Plaques were counted 21 days post infection.

We evaluated the inhibition of a GCV-resistant strain (EC_50_ of GCV = 7.6 µM) by dimers 832-4 and 838 using a plaque reduction assay. Dimers 832-4 and 838 were applied to infected cells at a concentration of 0.1 µM; GCV was applied at 5 µM and 30 µM. Twenty one days after infection, cells were stained with crystal violet and plaques were counted in each condition (Fig. 3). Dimers 832-4 and 838 fully inhibited plaque formation at 0.1 µM, while this strain showed an obvious resistance to GCV at 5 µM.

### Slopes of the anti-CMV dose response curves of the tested anti-CMV compounds

A classic description of dose-response relationships is the median effect model based on mass action indicating that [21]:
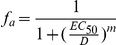
In this function, 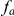 is the proportion of the inhibited fraction of viruses, or the inhibition percentage, *D* is drug concentration, *EC_50_* is the drug concentration that achieves 50% of the maximum inhibitory effect and *m* is a slope parameter of the curve mathematically. The effect of the slope parameter on anti-HIV activity was recently evaluated, and proved to be a characteristic of drug class and a crucial parameter dimension in the analysis of antiviral activity [21].

We applied this model to our derivatives and calculated the slope parameter of the most potent dimers (832-4 and 838), GCV, and artemisinin-derived monomer artesunate (Table 2). Compared to GCV and artesunate, which had an anti-CMV *m* value of approximately 1, the *m* values of 832-4, 838, 760 and 606 were around 4, an indication that these compounds are a highly potent class of anti-CMV compounds.

### Inhibition of cancer cell growth by dimers 832-4 and 838

Artemisinin-derived monomers and several dimers have been reported to inhibit the growth of cancer cell-lines [16], [17]. Four dimers (832-4, 838, 760 and 606), two monomers (artesunate and artefanilide) and GCV were applied to three cancer cell lines: HeLa (cervical adenocarcinoma), HCT116 (colorectal carcinoma) and 1205Lu (melanoma) at concentrations ranging from 0.01 µM to 1 µM. MTT assay was performed 72 hr after treatment and CC_50_ of each compound in each cancer cell line was calculated (table 3). The CC_50_ of each compound in cancer cells was compared to the CC_50_ in non-cancer primary HFF cells. All dimers were significantly more active than monomers in inhibiting the growth of cancer cell lines. For example, the dimer 838 was at least 200-fold more potent in inhibiting growth of HCT116 and 1205Lu cells and 37-fold more potent in inhibiting growth of HeLa cells, as compared to artesunate, the most potent monomer in this assay. A qualitative correlation was observed between the anti-CMV activity and the anti-cancer activity among the four dimers (838, 832-4, 606 and 760); dimer 838, the most potent of all tested dimers against CMV replication, also had the strongest effect on growth inhibition of cancer cells.

**Table 3:** CC_50_ in three types of cancer cell lines: HeLa, 1205Lu, HCT116, and in non-cancer primary HFF cells.

		CC_50_ (µM)		
Compound	HeLa	1205Lu	HCT116	HFF
832-4	0.27±0.03	0.13±0.007	0.07±0.006	57.5±2.9
838	0.19±0.08	0.06±0.002	0.04±0.005	55.8±2.8
760	0.56±0.18	0.1±0.006	0.07±0.005	4.9±0.2
606	1.06±0.2	4.9±0.04	0.1±0.001	48.1±2.6
Artesunate	7.1±0.4	42±2.4	11.3±0.6	71.7±4
Artefanilide	>50	>50	40±7.9	44.9±3.4
GCV	>50	>50	>50	247±33.4

## Discussion

We report here on the anti-CMV activity of ten new artemisinin-derived dimers, one deoxy dimer and one new artemisinin monomer, and the identification of the most potent anti-CMV dimer. The enhanced anti-CMV activity of dimers as compared to monomers was not a result of increased cytotoxcity but rather a specific anti-CMV activity. The two most highly potent dimers were effective in inhibition of the laboratory-adapted Towne strain as well as a GCV-resistant strain. They were also shown to have the strongest inhibitory effect on the growth of cancer cell lines.

All dimers exhibited potent CMV inhibition at 1 µM. However, only two compounds (832-4 and 838) could be selected as highly potent anti-CMV agents based on their high CMV inhibition at 0.1 µM and their low cytotoxicity. At this concentration, the other dimers had either no inhibition or at most 40% inhibition of luciferase expression, while GCV had no inhibition at all.

Artemisinin derivatives were initially developed as anti-malarial drugs, but later were also found to have additional pharmacological activities such as growth inhibition of cancer cells and inhibition of CMV replication [12], [13], [22]–[25]. Whether or not these new activities are related to each other and indicate a shared mechanism, the endoperoxide bridge appears to be critical in all pharmacological activities of artemisinin derivatives.

As a generalized observation, the dimers were significantly more potent than the monomers in CMV inhibition. Using the EC_50_ values_,_ dimer 838 was 165-fold more potent than artesunate in anti-CMV activity and its SI was 134-fold higher compared to artesunate. In support of this finding, the model of dose response slope parameter which was recently used to characterize the activities of anti-HIV compounds [21] revealed that dimers had a much higher slope than monomers. Generally, a higher slope correlates with a more vigorous anti-viral activity. This could result either from very high affinity of drug binding to a single ligand or from cooperative effect of multiple ligands with similar affinity.

Several anti-cancer agents (such as kinase inhibitors) have been shown to inhibit CMV replication, but in general, the concentrations required to achieve CMV inhibition were toxic to cells and prohibited their use as anti-viral candidates [26]. Interestingly, Sorafenib, a multi-kinase inhibitor, appears to inhibit CMV replication without associated cellular toxicity [27]. The dimers reported here prevented growth of cancer cell-lines and were potent inhibitors of CMV replication without apparent cellular toxicities. Therefore, they may provide a new class of anti-CMV agents, with a distinct mechanism of action. Similar to the anti-CMV activity, dimers were also more effective than monomers in inhibition of cancer cells. Future studies may reveal whether the dual anti-CMV and anti-cancer activities of dimers result from a shared mechanism of action. We and others have shown that inhibition of CMV replication with artemisinin derivatives appears early during the replication cycle and involves a mechanism which is different from the DNA polymerase inhibitors [28], [29]. The anti-cancer activities of artemisinins have been postulated to involve cell cycle arrest, apoptosis, and/or angiogenesis [30], processes that are also affected by specific CMV genes, mostly immediate early genes. Future studies will address the mechanism of CMV inhibition by artemisinin-derived dimers and their potential overlap with oncogenic signaling events.

In conclusion, we have shown that: 1). Dimers are highly active against CMV replication, and dimer diphenyl phosphate 838 is the most potent in CMV inhibition. 2). The dose response slope parameter of dimers is significantly higher than that of artemisinin monomers and GCV, a possible indication that dimers are a distinct class of anti-CMV compounds. 3) Dimers are more potent inhibitors than monomers in both cancer cell lines and in CMV.

## Materials and Methods

### Compounds

Ganciclovir (GCV) was obtained from Roche, USA. All artemisinin derivatives used in this study were synthesized at Johns Hopkins University [17], [31]–[33]. Newly synthesized dimer sulfide 3-carbamate (800-3) and dimer sulfide 4-carbamate (800-4) as well as dimer sulfone 3-carbamate (832-3) were synthesized as described in Materials S1. Artemisinin-derived monomers and dimers were dissolved in dimethyl sulfoxide (DMSO) and stocks of 10 mM were stored in −80°C. Synthetic compounds were at least 98% pure based on proton NMR spectroscopy. The DMSO itself was tested in CMV-infected cells, and it did not have any anti-viral activity [13].

### Viruses

The pp28-luciferase Towne strain was constructed as previously described [20]. Briefly, the recombinant virus, which expresses luciferase under the control of UL99 (pp28) late promoter was generated by insertion of the reporter gene between the US9 and US10 open reading frames (ORFs) in the Towne genome. The expression of pp28 –luciferase is strongly activated 48–72 hours post infection (hpi) and is almost completely inhibited in the presence of DNA polymerase inhibitors such as GCV and foscarnet [20]. We have recently reported that the pp28-luciferase reporter system is sensitive, reproducible and highly correlates with plaque reduction [20]. The ganciclovir (GCV)-resistant strain was obtained from a patient with CMV disease. It has a UL97 mutation (H520Q) and an EC_50_ of 7.6 µM for GCV. This clinical isolate was provided by the clinical virology laboratory with no identifiers that can link to a specific subject. The Johns Hopkins Office of Human Subject Research Institutional Review Board determined that this research qualified for an exemption.

### Cell Culture, Virus Infection and Anti-viral assays

Human Foreskin Fibroblasts (HFF) passage 12–16 (ATCC, CRL-2088™) were grown in Dulbecco's Modified Eagle Medium (DMEM) containing 10% fetal bovine serum (Gibco, Carlsbad, CA) in a 5% CO_2_ incubator at 37°C and used for infections with pp28-luciferase Towne CMV or the GCV-resistant strain. One day prior to infection with the pp28-luciferase, 4×10^4^ HFF cells were seeded on each well of 24-well tissue culture plates. Infection was carried out at multiplicity of infection of 1 PFU/cell (MOI = 1). Following 90 minute adsorption, media containing virus was removed and replaced by DMEM with 4% fetal bovine serum (Gibco, Carlsbad, CA) containing anti-viral compounds. The concentration of each compound was calculated and adjusted by volume such that it was constant throughout the experiment.

Infected and treated HFF cells were collected 72 hpi and washed once with PBS. The lysates were assayed for luciferase and cell viability using a Luciferase Assay Kit (Promega, Madison, WI) and CellTiter-Glo Luminescent Cell Viability Assay Kit, respectively, on GloMax®-Multi+ Detection System (Promega) according to manufacturer's instructions.

Human lung fibroblasts (HEL) passage 8–12 (ATCC, CCL-137™) were grown in DMEM containing 10% fetal bovine serum. One day prior to infection, 3×10^5^ cells were seeded on each well of 12-well tissue culture plates. GCV-resistant CMV was diluted in DMEM to a desired concentration which gave around 100 plaques per well and added to each well in duplicates. Plates were incubated for 90 minutes with shaking every 10 min; thereafter drugs were added and a methylcellulose overlay applied to each well. After incubation for 21 days, cells were stained with crystal violet and plaques were counted under microscope at 40× magnification.

### Anti-Cancer Cells Assay

HeLa (Human Cervical Adenocarcinoma), HCT116 (Human Colorectal Carcinoma) and 1205Lu (Human Melanoma) cells (all from ATCC) were maintained in DMEM containing 10% fetal bovine serum. 12–16 hours prior to drug treatment, 3×10^3^ to 5×10^3^ cells were seeded on each well of 96-well tissue culture plates. Drugs were diluted in DMEM containing 10% fetal bovine serum and applied to the cells. 72 hours after drug treatment, 20 µl of MTT solution (5 mg/ml in PBS) was added into each well, followed by shaking the plates at 150 rpm for 5 minutes and an incubation of 4 hours (37°C, 5% CO_2_). Media was removed, plates dried and each well was resuspended in 100 µl MTT formazan/DMSO. The absorbance of each well was recorded at 560 nm on GloMax®-Multi+ Detection System (Promega).

## Supporting Information

Materials S1 **1**) Synthesis of Dimer Sulfide 3-Carbamate **800-3. 2**) Synthesis of Dimer Sulfone 3-Carbamate **832-3. 3**) Synthesis of Dimer Sulfide 4-Carbamate **800-4. 4**) Synthesis of deoxy-artemisinin alcohol **574. 5**) Synthesis of Monomer Sulfone Benzylic Dimethyl Carbamate **551**.
(DOC)
