# Supplementary material for: An Artemisinin-Derived Dimer Has Highly Potent Anti-Cytomegalovirus (CMV) and Anti-Cancer Activities
Source: PLoS One. 2011 Aug 31;6(8):e24334. doi: 10.1371/journal.pone.0024334 (PMC3164168; doi:10.1371/journal.pone.0024334)
Supplement: Materials S1 — 1) Synthesis of Dimer Sulfide 3-Carbamate 800-3. 2) Synthesis of Dimer Sulfone 3-Carbamate 832-3. 3) Synthesis of Dimer Sulfide 4-Carbamate 800-4. 4) Synthesis of deoxy-artemisinin alcohol 574. 5) Synthesis of Monomer Sulfone Benzylic Dimethyl Carbamate 551. (DOC) [file pone.0024334.s001.doc]

Synthesis of Dimer Sulfide 3-Benzylic Alcohol

To a 2-dram vial equipped with stirbar under argon was added Cs_2_CO_3_ (0.076 g, 0.23 mmol, 1.7 eq) in anhydrous CH_3_CN (3.3 mL). A solution of 3-mercaptobenzyl alcohol (0.033 g, 0.23 mmol, 1.7 eq) in CH_3_CN (0.7 mL) was added dropwise. The reaction was allowed to stir for 30 minutes and then a solution of Br-Artemisinin dimer (0.090 g, 0.13 mmol, 1.0 eq) in CH_3_CN (2 mL) was added dropwise. The reaction was stirred at RT for 24 hours. At this time TLC analysis indicated complete reaction. The reaction was quenched with sat. NaHCO_3_, and the aqueous layer was extracted with CH_2_Cl_2_ (3x). The combined organics were dried over anhydrous MgSO_4_, filtered, and concentrated *in vacuo*. The resulting crude material was purified via column chromatography (30% EtOAc in hexanes) to yield the desired sulfide (0.085 g, 0.12 mmol) as a colorless oil in 89% yield.

^1^H NMR (400 MHz, CD_3_Cl) δ 7.44 (s, 1H); 7.29-7.26 (t, 1H, J = 7.6 Hz); 7.20-7.16 (t, 1H, J = 15.2 Hz); 7.10-7.08 (d, 1H, J = 7.6 Hz); 5.23 (s, 1H); 5.15 (s, 1H); 4.6 (s, 2H)4.35-4.31 (m, 1H); 4.18-4.14 (m, 1H); 3.45-3.34 (m, 1H); 3.09-3.07 (m, 1H); 2.66-2.64 (m, 1H); 2.41-2.39 (m, 1H)2.28-2.25 (m, 3H); 2.05-1.55 (12H); 1.38 (s, 3H); 1.34 (s, 3H); 0.92-0.7 (15H) ^13^C NMR (100 MHz, CD_3_Cl) δ 171.0, 142.0, 137.3, 128.7, 128.6, 127.8, 124.4, 103.3, 102.9, 89.4, 88.6, 81.2, 81.1, 74.1, 71.0, 65.0, 64.2, 60.3 ,52.4, 52.1, 44.6, 44.1, 37.5, 37.3, 37.2, 36.6, 36.5, 34.4, 34.3, 33.6, 31.8, 31.4, 30.5, 30.4, 30.3, 26.0, 25.9, 24.8, 24.6, 24.5 IR (cm^-1^) 3474 (b), 2937, 2874, 2361, 1592, 1450, 1376, 1007

[α]_D_^22.7^ = 48.4 (c = 0.68, CHCl_3_) HRMS calc for C_41_H_60_O_9_SNa+ (M+Na+) 751.3865, found 751.3868

Synthesis of Dimer Sulfide 3-Carbamate **800-3**

To a 2-dram vial equipped with stirbar under argon was added Artemisinin-dimer-sulfide benzyliz alcohol (0.016 g, 0.02 mmol, 1.0 eq) in CH_3_CN (1 mL). NaH (0.5 mg, 0.02 mmol, 1.0 eq) was added, immediately followed by dimethylcarbamoyl chloride (4.3 mg, 0.04 mmol, 2.0 eq). Reaction was stirred at room temperature overnight. At this time TLC analysis indicated complete consumption of the starting material. The reaction was quenched with sat. NaHCO_3_ and the aqueous layer was extracted with Et_2_O (3x). The combined organics were dried over anhydrous MgSO_4_, filtered, and concentrated in vacuo. Resulting crude oil was purified via column chromatography to give sulfide 3-carbamate **800-3** (0.012 g, 0.015 mmol) as a colorless oil in 75% yield.

^1^H NMR (400 MHz, CD_3_Cl) 7.37-7.35 (m, 2H); 7.25-7.21 (m, 1H); 7.13-7.11 (d, 1H, J = 7.2 Hz); 5.32 (s, 1H); 5.29 (s, 1H); 5.06 (s, 2H); 4.366-4.32 (m, 1H); 4.23-4.19 (m, 1H); 3.32-3.29 (m, 1H); 3.22-3.17 (m1, 1H); 2.93 (s, 6H); 2.70-2.68 (m, 1H); 2.54-2.52 (m, 1H); 2.34-2.27 (m, 3H); 2.0-1.5 (18H); 1.39 (s, 3H); 1.38 (s, 3H); 1.3-0.74 (23H)

^13^C NMR (100 MHz, CD_3_Cl) 137.7, 137.5, 128.8, 128.7, 128.6, 125.0, 103.2, 102.9, 89.3, 88.7, 81.2, 76.6, 74.2, 71.8, 66.7, 52.5, 52.2, 44.6, 44.3, 38.0, 37.4, 37.3, 36.6, 34.7, 34.5, 34.4, 31.9, 31.7, 30.5, 30.4, 26.1, 26.0, 24.8, 24.7, 27.6, 24.5 IR (cm^-1^) 3582, 2935, 1707, 1450, 1376, 1185, 1102, 1055, 1008 [α]_D_^22.7^ = 39.9 (c = 0.70, CHCl_3_) HRMS calc for C_44_H_65_NO_10_SNa+ (M + Na+) calc. 822.4221 found 822.4237

Synthesis of Dimer Sulfone 3-Carbamate **832-3**

To a 2-dram vial equipped with stirbar under argon was added Artemisinin-dimer-sulfide benzylic alcohol (0.045 g, 0.060 mmol, 1.0 eq) in CH_2_Cl_2_ (3 mL). *m*-CPBA (0.027 g, 0.12 mmol, 2.0 eq) was added and reaction was stirred at RT for 2 hours. At this time TLC analysis indicated complete consumption of the starting material and the reaction was quenched with sat. sodium bisulfate solution. The aqueous layer was extracted with CH_2_Cl_2_ (2x). The combined organic layers were washed with sat. NaHCO_3_, dried over anhydrous MgSO_4_, filtered, and concentrated *in vacuo*. The resulting crude dimer sulfone benzylic alcohol was used as is without further purification for the next reaction.

To a 2-dram vial equipped with stirbar under argon was added Artemisinin-dimer-sulfone benzylic alcohol (0.022 g, 0.029 mmol, 1.0 eq) in CH_3_CN (1 mL). NaH (0.7 mg, 0.029 mmol, 1.0 eq) was added, immediately followed by dimethylcarbamoyl chloride (6.2mg, 0.058 mmol, 2.0 eq). Reaction was stirred at room temperature overnight. At this time TLC analysis indicated complete consumption of the starting material. The reaction was quenched with sat. NaHCO_3_ and the aqueous layer was extracted with Et_2_O (3x). The combined organics were dried over anhydrous MgSO_4_, filtered, and concentrated in vacuo. Resulting crude oil was purified via column chromatography to give dimer sulfone 3-carbamate **832-3** (0.016 g, 0.019 mmol) as a colorless oil in 67% yield over two steps.

^1^H NMR (400 MHz, CD_3_Cl) 7.99-7.97 (d, 1H, J = 8Hz); 7.94 (s, 1H); 7.63-7.61 (d, 1H, J = 7.6 Hz); 7.53-7.49 (t, 1H, J = 7.6 Hz); 5.47 (s, 1H); 5.33 (s, 1H); 5.18 (s, 2H); 4.48-4.40 (m, 1H); 4.19-4.10 (m, 1H); 3.63-3.560 (dd, 1H, J = 9.21 Hz, 14 Hz); 3.38-3.37 (m, 1H); 2.94 (s, 6H); 2.68-2.66 (m, 1H); 2.55-2.52 (m, 2H); 2.4-0.7 (45H)

^13^C NMR (100 MHz, CD_3_Cl) 156.1, 140.6, 138.3, 132.5, 129.3, 127.7, 127.2, 103.3, 102.8, 89.5, 88.8, 81.3, 81.2, 76.6, 73.7, 70.7, 66.0, 58.9, 52.5, 52.1, 44.5, 44.0, 37.4, 37.3, 36.6, 35.9, 34.5, 34.4, 31.1, 31.0, 30.9, 30.7, 30.3, 26.2, 26.0, 24.7, 24.6, 23.3, 20.2

IR (cm^-1^) 3582, 2924, 2358, 1700, 1450, 1377, 1300, 1188, 1140, 1054, 1008

[α]_D_^23.8^ = 52.1 (c = 0.75, CHCl_3_) HRMS calc for C_44_H_65_NO_12_SNa+ (M+Na+) 854.4120 found. 854.4134

Synthesis of Dimer Sulfide 4-benzylic alcohol

To a 2-dram vial equipped with stirbar under argon was added Cs_2_CO_3_ (0.038 g, 0.11mmol, 1.7 eq) in anhydrous CH_3_CN (1.5 mL). A solution of 4-mercaptobenzyl alcohol (0.017 g, 0.11 mmol, 1.7 eq) in CH_3_CN (0.5 mL) was added dropwise. The reaction was allowed to stir for 30 minutes and then a solution of Br-Artemisinin dimer (0.045 g, 0.07 mmol, 1.0 eq) in CH_3_CN (1.5 mL) was added dropwise. The reaction was stirred at RT for 24 hours. At this time TLC analysis indicated complete reaction. The reaction was quenched with sat. NaHCO_3_, and the aqueous layer was extracted with CH_2_Cl_2_ (3x). The combined organics were dried over anhydrous MgSO_4_, filtered, and concentrated *in vacuo*. The resulting crude material was purified via column chromatography (30% EtOAc in hexanes) to yield the desired sulfide dimer (0.016 g, 0.02 mmol) as a colorless oil in 34% yield.

^1^H NMR (400 MHz, CD_3_Cl) δ 7.41-7.39 (d, 2H, 8Hz); 7.25-7.35 (d, 2H, J = 8Hz); 5.32 (s, 1H); 5.24 (s, 1H); 4.64 (s, 2H); 4.36-4.32 (m, 1H); 4.21-4.17 (m,1H); 4.06-4.04 (m,1H); 3.33-3.29 (dd, 1H, J = 4 Hz, J = 12 Hz); 3.17-3.12 (dd, 1H, J = 7.2 Hz, 13.2 Hz); 2.72-2.68 (m, 1H); 2.58-2.52 (m, 1H); 2-0.8 (45H) ^13^C NMR (100 MHz, CD_3_Cl) δ 138.3, 136.6, 129.7, 127.6, 103.3, 102.9, 89.3, 88.7, 21.2, 74.2, 71.8, 65.8, 65.0, 52.5, 52.2, 44.6, 44.3, 38.1, 37.4, 37.3, 36.6, 36.5, 34.5, 34.4, 34.3, 31.7, 30.5, 30.4, 26.1, 26.0, 24.8, 24.7, 24.6, 20.2 IR (cm^-1^) 3450 (b), 2950, 2874, 1683, 1492, 1450, 1376, 1278, 1091, 1052, 1007

[α]_D_^21.6^ = 55.1 (c = 0.8, CHCl_3_) HRMS calc for C_41_H_60_O_9_SNa+ (M+Na+) 751.3865 found 751.3865

Synthesis of Dimer Sulfide 4-Carbamate **800-4**

To a 2-dram vial equipped with stirbar under argon was added Artemisinin-dimer-sulfide para-benzylic alcohol (0.016 g, 0.02 mmol, 1.0 eq) in CH_3_CN (1 mL). NaH (0.5 mg, 0.02 mmol, 1.0 eq) was added, immediately followed by dimethylcarbamoyl chloride (4.3 mg, 0.04 mmol, 2.0 eq). Reaction was stirred at room temperature overnight. At this time TLC analysis indicated complete consumption of the starting material. The reaction was quenched with sat. NaHCO_3_ and the aqueous layer was extracted with Et_2_O (3x). The combined organics were dried over anhydrous MgSO_4_, filtered, and concentrated in vacuo. Resulting crude oil was purified via column chromatography to give sulfide 4-carbamate **800-4** (0.012 g, 0.015 mmol) as a colorless oil in 75% yield.

^1^H NMR (400 MHz, CD_3_Cl) 7.34-7.33 (d, 2H, J = 16 Hz); 7.22-7.19 (d, 2H, J = 16Hz); 5.32 (s, 1H); 5.29 (s, 1H); 5.04 (s, 2H); 4.40-4.41 (m, 1H); 4.34-4.28 (m, 1H); 3.50-3.45 (q, 2H, J = 7.2, J = 14 Hz); 3.31-3.25 (m, 1H); 3.21-3.0 (3H); 2.92 (6H); 2.75-2.66 (m, 1H); 2.61-2.53 (m, 1H); 2.3-0.8 (41H) ^13^C NMR (100 MHz, CD_3_Cl) 137.3, 134.1, 129.2, 128.4, 103.2, 102.9, 89.2, 88.7, 81.2, 74.2, 72.0, 66.7, 65.8, 52.5, 52.3, 44.6, 44.3, 38.0, 37.4, 37.3, 36.6, 34.8, 34.5, 31.8, 31.6, 30.5, 30.4, 26.2, 26.1, 24.8, 24.7, 24.6, 24.5, 20.2 IR (cm^-1^) 3608, 3582, 2923, 2873, 1707, 1494, 1450, 1182, 1101, 1009

[α]_D_^22.7^ = 48.4 (c = 0.55, CHCl_3_) HRMS calc for C_44_H_65_NO_10_SNa+ (M + Na+) calc. 822.4221 found 822.4228

Synthesis of deoxy-artemisinin dimer alcohol 574

Synthesis of deoxy-artemisinin – To a stirring solution of artemisinin (112.5 mg, 0.395 mmol, 1.0 eq) in acetic acid (AcOH, 10 mL) was added activated zinc (Zn, 218 mg, 3.33 mmol, 8.4 eq). The reaction mixture was stirred at room temperature for 2 h, at which time it was filtered through celite and diluted with chloroform. The organic layer was washed with water and sat. aq. sodium bicarbonate. The organic layer was extracted, dried over MgSO_4_, filtered and concentrated under reduced pressure. The residue was purified directly on silica. Gradient elution (10-20% ethyl acetate in hexanes) afforded the desired product as a colorless amorphous solid: yield (80 mg, 0.30 mmol, 76%); ^1^H NMR (400 MHz, CDCl_3_) δ 5.66 (s, 1 H), 3.15 (qd, *J* = 7.20, 4.59 Hz, 1 H), 2.02 – 1.93 (m, 1 H), 1.92 - 1.83 (m, 2 H), 1.80 – 1.70 (m, 2 H), 1.65 - 1.54 (m, 1 H), 1.51 (s, 3 H), 1.29 - 1.20 (m, 3 H), 1.16 (d, *J* = 7.25 Hz, 3 H), 1.10 – 0.96 (m, 2 H), 0.94 - 0.88 (m, 3 H).

Synthesis of deoxy-dihydroartemisinin acetate – A solution of deoxy-artemisinin (50 mg, 0.19 mmol, 1.0 eq) in 3 mL dichloromethane (DCM) was cooled to -78 ^o^C in a dry ice/acetone bath for 20 mins, at which time diisobutyl aluminum hydride (DIBALH, 1.0M in DCM, 0.23 mL, 0.23 mmol, 1.2 eq). The reaction mixture was stirred at -78 ^o^C for 1.5h, at which time total consumption of starting material was observed by TLC. Dimethylamino pyridine (DMAP, 27.5 mg, 0.23 mmol, 1.2 eq), pyridine (py, 0.053 mL, 0.66 mmol, 3.5 eq), and acetic anhydride (Ac_2_O, 0.089 mL, 0.94 mmol, 5.0 eq) were subsequently added in that order, and the reaction was warmed to rt and stirred overnight. Upon completion as observed by TLC, the reaction was diluted with EtOAc and washed with 2x 5% citric acid, sat. NH_4_Cl, and brine. The organic layer was dried over MgSO_4_, filtered, and the solvent was removed under reduced pressure. The crude residue was purified directly on silica. Gradient elution (3 – 10% EtOAc in hexanes) afforded the desired product as a colorless, amorphous solid: yield (33.0 mg, 0.106 mmol, 57%); ^1^H NMR (400 MHz, CDCl_3_) δ 5.72 (d, *J* = 6.26 Hz, 1 H), 5.31 (s, 1 H), 2.57 - 2.44 (m, 1 H), 2.07 (s, 3 H), 1.87 - 1.78 (m, 3 H), 1.73 (dd, *J* = 15.02, 2.12 Hz, 2 H), 1.67 - 1.57 (m, 2 H), 1.55 - 1.50 (m, 3 H), 1.30 - 1.18 (m, 4 H), 0.96 (d, *J* = 7.35 Hz, 3 H), 0.89 (d, *J* = 5.56 Hz, 3 H).

Synthesis of deoxy-artemisinin isobutylene dimer – To a 2 dram vial were added deoxy-artemisinin acetate (27.3 mg, 0.088 mmol, 1.0 eq), (2-methylenepropane-1,3-diyl)bis-trimethylsilane (15.0 mg, 0.075 mmol, 0.85 eq), and DCM (1.5 mL). The stirring solution was cooled to -78 ^o^C in a dry ice/acetone bath for 20 mins, at which time tin (IV) chloride (SnCl_4_, 1.0 M in DCM, 0.089 mmol, 1.01 eq, further diluted to 1 mL) was added dropwise over several minutes. The reaction mixture was allowed to stir for 2h, at which time the reaction was diluted with DCM and quench with water. The organic layer was extracted without further workup, dried over MgSO_4_, filtered, and the solvent was removed under reduced pressure. The crude residue was purified directly on silica. Gradient elution (2 – 10% EtOAc in hexanes) afforded the desired product as a colorless, amorphous solid: yield (12.0 mg, 0.021 mmol, 45%); ^1^H NMR (400 MHz, CDCl_3_) δ 5.26 (s, 2 H), 4.97 (s, 2 H), 3.60 (td, *J* = 8.82, 3.13 Hz, 2 H), 2.45 (dd, *J* = 14.39, 2.66 Hz, 2 H), 2.32 - 2.14 (m, 4 H), 1.81 - 1.68 (m, 10 H), 1.65 (d, *J* = 5.84 Hz, 3 H), 1.58 - 1.47 (m, 9 H), 1.23, 1.40 (m, 9 H), 1.23 - 1.2 (m, 5 H), 1.11 – 0.99 (m, 4 H), 0.92 - 0.82 (m, 13 H).

Synthesis of deoxyartemisinin isobutylene dimer alcohol 574 – To a 2 dram vial were added deoxy-artemisnin isobutylene dimer (12.0 mg, 0.021 mmol, 1.0 eq), and tetrahydrofuran (THF, 2 mL). The stirring solution was cooled to 0 ^o^C for 20 mins, at which time borane-dimethyl sulfide complex (BH_3_:DMS, 2.0 M in THF, 0.013 mL, 1.2 eq) and the reaction mixture was warmed to rt and allowed to stir for 3h. At that time, a slurry of sodium perborate tetrahydrate (NaBO_3_:4 H_2_O, 24 mg, 0.108 mmol, 5.0 eq) was carefully added. The reaction mixture was stirred for another 30 mins, then diluted with EtOAc and washed several times with water. The organic layer was dried over MgSO_4_, filtered and the solvent was removed under reduced pressure. The crude residue was purified directly on silica. Gradient elution (10 – 20% EtOAc in hexanes afforded the desired product as a colorless, amorphous solid: yield (9.5 mg, 0.017 mmol, 77%); ^1^H NMR (400 MHz, CDCl_3_) δ 5.24 (s, 2 H), 3.71 – 3.56 (m, 4 H), 3.52 – 2.43 (m, 2 H), 2.23 – 2.05 (m, 5 H), 1.81 – 1.69 (m, 10 H), 1.67 – 1.59 (m, 5 H), 1.57 – 1.48 (m, 8 H, including singlets at 1.52 and 1.50), 1.40 – 1.15 (m, 8 H), 0.92 – 0.86 (d, 4 H, *J* = 5.7 Hz), 0.85 – 0.80 (d, 6 H, *J* = 6 Hz); ^13^C NMR (100 MHz, CDCl_3_) δ 108.1, 107.9, 96.0, 95.9, 83.1, 82.6, 74.5, 72.4, 64.9, 46.5, 46.3, 42.5, 42.3, 37.8, 36.6, 35.7, 35.2, 35.1, 34.6, 34.6, 34.4, 34.4, 31.6, 30.7, 24.7, 24.5, 24.5, 22.5, 22.1, 19.0, 19.0, 15.5, 14.8; HRMS (ESI) m/z for C_34_H_54_O_7_ (M + Na)^+^ calcd = 597.3762, found = 597.3753; [α]_D_^23^ = -77 (c = 0.20, CHCl_3_).

**Synthesis of Monomer Primary Alcohol**

To a solution of allyl monomer (373 mg, 1.2 mmol) in THF (10 mL) at 0 ºC was added a 2.0 M in diethyl ether solution of borane-methyl sulfide complex (730 μL, 1.45 mmol) in THF (5 mL) *via* canula. The solution was allowed to stir for 4 h. A solution of sodium perborate tetrahydrate (750 mg, 4.9 mmol) in distilled water (10 mL) was added to the reaction and allowed to stir at room temperature overnight. The organic layer was extracted with dichloromethane (2 x 10 mL). The organic layer was dried with MgSO_4_, filtered, and concentrated *in vacuo*. The crude product was purified by flash silica gel column chromatography (40% EtOAc/hexanes) to yield **monomer primary alcohol** (300 mg, 0.92 mmol, 76%) as a colorless oil. The data match those reported in the literature.

**Synthesis of Monomer Primary Bromide**

To a room temperature solution of monomer primary alcohol (100 mg, 0.31 mmol) in dichloromethane (5 mL) was added triphenylphosphine (121 mg, 0.46 mmol) and carbon tetrabromide (153 mg, 0.46 mmol). The solution was allowed to stir at room temperature for 1 h. The reaction was quenched with brine (5 mL) and the organic layer was extracted with dichloromethane (2 x 5 mL). The organic layer was dried with MgSO_4_, filtered, and concentrated *in vacuo*. The crude product was purified by flash silica gel column chromatography (20% EtOAc/hexanes) to yield **monomer primary bromide** (103 mg, 0.83 mmol, 83%) as a sticky solid. [α]_D_^21.3^ +71.2° (c = 0.48, CHCl_3_); IR (thin film) 2953, 2875, 1450, 1375, 1278, 1251, 1222, 1205, 1116, 1091, 1056, 1009, 946, 913, 877 cm^-1^; ^1^H NMR (400 MHz, CDCl_3_) δ 5.28 (s, 1H), 4.19-4.14 (m, 1H), 3.51-3.46 (m, 2H), 2.67-2.62 (m, 1H), 2.43-2.26 (m, 1H), 2.18-2.14 (m, 1H), 2.04-1.22 (16H including a singlet at 1.39 for 3H), 0.95-0.93 (d, J = 6.0 Hz, 4H), 0.87-0.85 (d, J = 7.6 Hz, 3H); ^13^C NMR (100 MHz, CDCl_3_) δ 103.1, 89.1, 81.1, 74.4, 52.3, 44.3, 37.5, 36.6, 34.4, 34.3, 30.6, 30.3, 27.8, 26.1, 24.9, 24.7, 20.2, 12.9; HRMS (FAB) *m/z* calcd for C_18_H_30_BrO_4_ (M+H)^+^ 391.1307, found 391.1296.

**Synthesis of Monomer Sulfone 4-Benzylic Alcohol**

To a solution of monomer primary bromide (100 mg, 0.25 mmol) in anhydrous acetonitrile (5 mL) was added 4-hydroxymethyl thiophenol (100 mg, 0.50 mmol, prepared by LAH reduction of the commercially available acid). NaH (20 mg, 0.83 mmol) was added to the solution (gas evolution) and was allowed to stir at room temperature for 1 h. The reaction was quenched with sat. aq. sodium bicarbonate (5 mL) and extracted with dichloromethane (2 x 5 mL). The organic layer was dried with MgSO_4_, filtered, and concentrated *in vacuo*. The crude oil was dissolved in dichloromethane (5 mL) and the sulfide was oxidized at room temperature to the sulfone with mCPBA (213 mg, 1.2 mmol) over 2 h. The reaction was quenched with saturated aqueous sodium bisulfite (5 mL) and the organic layer was extracted with sat. aq. sodium bicarbonate (5 mL). The crude product was purified by flash silica gel column chromatography (40🡪 50% EtOAc/hexanes) to yield **monomer sulfone 4-benzylic alcohol** (69 mg, 0.14 mmol, 58%) as an amorphous white solid. [α]_D_^21.2^ +50.3° (c = 0.48, CHCl_3_); IR (thin film) 3520 (br), 2970, 2944, 2876, 1452, 1409, 1379, 1301, 1221, 1204, 1143 (s), 1088, 1055, 1013, 946, 876, 824, 756, 669 cm^-1^; ^1^H NMR (400 MHz, CDCl_3_) δ 7.87-7.85 (d, J = 8.4 Hz, 2H), 7.54-7.52 (d, J = 8.4 Hz, 2H), 5.25 (s, 1H), 4.79 (s, 2H), 4.14-4.09 (m, 1H), 3.22-3.18 (t, J = 7.6 Hz, 2H), 2.59-2.54 (m, 1H), 2.34-2.26 (m, 1H), 2.04-1.18 (m, 17H including a singlet at 1.37 for 3H), 0.95-0.94 (d, J = 6.0 Hz, 4H), 0.81-0.79 (d, J = 7.6 Hz, 3H)HHHH; ^13^C NMR (100 MHz, CDCl_3_) δ 147.4, 138.0, 128.3, 127.1, 103.1, 89.3, 81.1, 74.2, 64.2, 55.8, 52.2, 44.1, 37.5, 36.6, 34.4, 30.3, 27.9, 26.1, 24.8, 24.7, 21.2, 20.1, 12.7; HRMS (ESI) *m/z* calcd for C_25_H_36_O_7_SNa (M+Na)^+^ 503.2074, found 503.2065.

**Synthesis of Monomer Sulfone 4-Benzylic Dimethyl Carbamate 551**

To a solution of monomer sulfone benzylic alcohol (40 mg, 0.083 mmol) in DMF (2 mL) was added dimethylcarbamyl chloride (15 μL, 0.17 mmol) and NaH (4 mg, 0.17 mmol). The reaction was stirred for 30 min and quenched with sat. aq. sodium bicarbonate (2 mL). The organic layer was extracted with dichloromethane (2 x 5 mL) and then dried with MgSO_4_, filtered, and concentrated *in vacuo*. The crude product was purified by flash silica gel column chromatography (30🡪 50% EtOAc/hexanes) to yield **monomer sulfone 4-benzylic dimethyl carbamate** (41 mg, 0.074 mmol, 89%) as an amorphous white solid. [α]_D_^21.8^ +55.6° (c = 0.25, CHCl_3_); IR (thin film) 2972, 2943, 2877, 1707 (s), 1496, 1451, 1395, 1357, 1306, 1208, 1180, 1144 (s), 1119, 1088, 1057, 1010, 769 (s) cm^-1^; ^1^H NMR (400 MHz, CDCl_3_) δ 7.89-7.87 (d, J = 8.4 Hz, 2H), 7.52-7.50 (d, J = 8.4 Hz, 2H), 5.25 (s, 1H), 5.18 (s, 2H), 4.14-4.09 (m, 1H), 3.22-3.18 (t, J = 8.4 Hz, 2H), 2.95 (s, 6H), 2.59-2.54 (m, 1H), 2.33-2.25 (m, 1H), 2.03-1.17 (m, 17H including a singlet at 1.36 for 3H), 0.94-0.92 (d, J = 6.0 Hz, 4H), 0.81-0.79 (d, J = 7.6 Hz, 3H); ^13^C NMR (100 MHz, CDCl_3_) δ 156.0, 143.2, 138.6, 128.4, 128.1, 103.0, 89.3, 81.1, 74.1, 65.8, 55.8, 52.2, 44.1, 37.5, 36.6, 34.4, 30.3, 27.9, 26.1, 24.8, 24.7, 21.1, 20.1, 12.7; HRMS (ESI) *m/z* calcd for C_28_H_41_NO_8_SNa (M+Na)^+^ 574.2445, found 574.2450.
